# Occurrence of cardiorespiratory diseases and impact on lifespan in Swedish Irish Wolfhounds: a retrospective questionnaire-based study

**DOI:** 10.1186/s13028-017-0320-1

**Published:** 2017-08-02

**Authors:** Lovisa Orleifson, Ingrid Ljungvall, Katja Höglund, Jens Häggström

**Affiliations:** 1 Department of Clinical Sciences, Faculty of Veterinary Medicine, Swedish University of Agricultural Sciences, Uppsala, Sweden; 2 Department of Anatomy, Physiology and Biochemistry, Faculty of Veterinary Medicine, Swedish University of Agricultural Sciences, Uppsala, Sweden

**Keywords:** Dilated cardiomyopathy, Irish Wolfhound, Lifespan, Mortality, Pneumonia

## Abstract

**Background:**

According to Swedish animal insurance data, Irish Wolfhounds (IW) are 29 times more likely to die from cardiac causes than the baseline breed. Dilated cardiomyopathy (DCM) has a high prevalence in the breed and the disease has been shown to be hereditary in IW. Few studies address respiratory diseases in IW, but reports suggest that the incidence of pneumonia is high. Respiratory diseases are reported as a common cause of death in the breed along with cardiac, neoplastic, musculoskeletal and gastrointestinal diseases. The aim of this study was to investigate mortality, morbidity and lifespan in Swedish IW through a questionnaire-based study. Focus was on DCM and pneumonia and potential association between these diseases. Questionnaires were sent to owners of purebred IW registered in the Swedish Kennel Club, born during 2006–2008. Owners were asked for information concerning occurrence of disease, results of clinical examinations, treatments, cause and date of death.

**Results:**

Overall response rate was 38% (105 completed questionnaires). Median lifespan was 2720 days (7.5 years). Males had shorter lifespan than females (median 2523 and 2836 days, respectively), P = 0.02. The most common causes of death were neoplastic disease (24%), cardiac disease (18%) and respiratory disease (16%). The percentage of dogs with pneumonia on at least one occasion during their lifetime was 37%, with a majority experiencing recurrent episodes (53%). The median lifespan was shorter for dogs affected by pneumonia on at least one occasion (2629 days), compared to dogs without history of pneumonia (2804 days) (P = 0.04), whereas the lifespan did not differ between dogs with or without a diagnosis of DCM. No sex predisposition was found regarding DCM or pneumonia.

**Conclusions:**

This study showed that DCM and pneumonia are common conditions in IW in Sweden, and that dogs affected by pneumonia have a shorter lifespan than those without history of pneumonia. Considering the results from this study and previous studies regarding these diagnoses in IW; cardiac and respiratory disease should be given further attention in the course of improving the general health of the breed.

**Electronic supplementary material:**

The online version of this article (doi:10.1186/s13028-017-0320-1) contains supplementary material, which is available to authorized users.

## Background

Irish Wolfhounds (IW) have an increased mortality because of neoplastic, musculoskeletal, gastrointestinal, cardiovascular and respiratory disorders [1–3]. Based on Swedish insurance data, the breed had the highest probability of death by 8 and 10 years of age, compared to all other breeds in the insurance database, which included over 350,000 insured Swedish dogs [1]. Indeed, the probability of death by 10 years of age was as high as 91% in IW. The median and mean age at time of death for IW studied in Great Britain and Norway were 6.2 and 7 years, respectively [2, 4].

According to Swedish insurance data, IW were 29 times more likely to die from cardiac disorders, compared to the baseline breeds defined as and including all breeds, except 10 high risk breeds and the 10 most common breeds [1]. Dilated cardiomyopathy (DCM) is a common disease in European IW [5, 6] and is characterized by uni- or bilateral cardiac dilation and reduced systolic function [7]. Generally, DCM in dogs progresses quickly and overall prognosis is poor [8]. However, a difference in progression rate between breeds has been shown [9–11]. Swedish insurance data has shown that median age at death in IW with cardiac disease was 7.5 years [12]. In European studies, the prevalence of DCM among IW has been found to be 24–43% [5, 6, 13], where mean age at diagnosis was 4.2–4.9 years [5, 6, 14]. Genetic factors have been shown to influence the development of DCM in several breeds [10, 15, 16]. Regarding mode of inheritance in IW, a major gene model with a sex-dependent allele effect has been suggested [6], and another study suggested that multiple loci were potentially associated with DCM in this breed [14].

Few reports regarding respiratory diseases in IW are available, but, anecdotally, IW are considered more frequently affected by pneumonias than most other breeds. In a recent study, determining the incidence of pneumonia in dogs admitted to a veterinary hospital, IW had the highest incidence of all breeds [17]. A study describing rhinitis and pneumonia in the breed showed that the majority of affected dogs had clinical signs present since birth [18]. Pedigree analysis revealed that ancestors were shared, which according to the authors, suggested a heritable syndrome. Primary ciliary dyskinesia or primary immunodeficiency were not detected among the affected dogs and signs of cardiac disease were also absent. Rhinitis and pneumonia in IW was early described by Wilkinson [19] who rather suggested the etiology to be a viral infection with secondary bacterial invasion due to the condition being well distributed throughout the breed.

Although insurance data are a valuable source of information to evaluate morbidity and causes of death in specific dog breeds, the resolution of the information is limited and the database does not include old dogs. The aim of this study was, therefore, to investigate mortality, morbidity and lifespan in Swedish IW through a questionnaire-based study, with special focus on DCM and pneumonia and potential associations between the diseases, using the Swedish Kennel Club (SKK) registration database as source of dog and owner related information.

## Methods

### Study design

This retrospective study was performed by distributing a questionnaire (Additional file 1: Questionnaire) to owners of purebred IW born between January 1st 2006 and December 31st 2008 registered in the SKK (460 dogs). The questionnaire was sent by mail to all owners with an address available in the SKK register (n = 280) and since several dogs shared the same owner 280 questionnaires were sent to 232 owners. Recipients that had not responded within the stipulated time received one reminder. Information about the study and reminding notices were published at the webpage and the Facebook page of the Swedish Irish Wolfhound Club. The questionnaire was referred to as a health evaluation of Swedish IW and owners with healthy dogs as well as sick dogs were encouraged to answer.

The questionnaire was divided into three sections, where the first, which was for everyone to answer, covered general information such as sex, identification number, neutering status, weight, coat color, breeding history and the owners contact information. The second section was for dogs that were dead, and the third section for dogs that were still alive. The second section contained questions about cause of death, divided into different main classes, date of death, potential history of DCM or pneumonia, clinical signs observed by the owner and if a post mortem examination had been performed. Questions also concerned medical treatment for DCM and if pneumonias were recurrent. In the third section, there were questions concerning history of DCM or pneumonia, clinical signs observed by the owner and more specific questions whether heart and thorax of the dog had been examined by a veterinarian, and if the dog was subject to ongoing medical treatment as well as if potential pneumonias were recurrent. For all dogs, questions were also asked if known relatives were affected by DCM or pneumonia. In this study, relatives were defined as mother, father, siblings and offspring. The information from the questionnaires was treated confidentially and permission was asked for access to veterinary records if considered required.

### Data management

Complete and correct birth date, as well as sex and coat color, were verified for each dog by use of the SKK register. For owners that had not reported date of death as an exact date, but as a month or a year, date of death was set to the middle of the reported period. Deceased dogs with no information of date of death were excluded from the survival analyses.

Information about clinical signs and results from examinations, that were associated with cardiac disease, were evaluated to determine whether the evidence was sufficiently strong to allow a diagnosis of DCM or not: dogs, for which the owner had reported DCM and/or the diagnosis of DCM had been established by echocardiographic examinations, or a post mortem examination, were classified as DCM. Dogs with other abnormal findings, such as atrial fibrillation, low intensity heart murmur, and/or having developed congestive heart failure (CHF) were classified as “probably” DCM. Dogs diagnosed with CHF at time of euthanasia, but not having undergone more detailed diagnostic procedures, with no prior clinical signs of cardiac disease, were classified as “suspicion of DCM”. Dogs with DCM, with suspicion of DCM and those probably affected were all later included when calculating occurrence and lifespan regarding DCM.

### Statistical analyses

The statistical analyses were performed using a commercially available statistical software program (JMP Pro v. 11.2.0, Cary, NC, USA). Level of statistical significance was set at P < 0.05. Descriptive statistics were used for dog characteristics, causes of death and occurrence of diagnoses such as DCM and pneumonia. If owners had reported multiple causes of death for one dog, the dog was included in each subgroup in the statistical analysis. Data concerning dogs from the same owner was not clustered in the statistical analysis.

Fisher’s exact test was used to test for differences in occurrence of DCM and pneumonia between sexes. Kaplan–Meier curves were generated and the Log Rank test was used to test for difference in lifespan between subgroups in the study population. Lifespan for dogs that were still alive at the time of this study were right censored. Lifespan was reported as median and interquartile range for the whole study population and for subgroups. Multivariable Cox proportional hazard analysis was used to investigate the effect of sex and diagnosis on outcome and for calculating the hazard ratio, variables included in these models were sex and occurrence of pneumonia on at least one occasion, and sex and diagnosis of DCM, respectively.

## Results

Of the 280 distributed questionnaires, 105 were completed and returned by mail to the Swedish University of Agricultural Sciences (SLU). Four letters were undelivered and returned to SLU because of incorrect contact details. This resulted in a total response rate of 38%. In total, 90 owners participated in the study; 80 owners returned one questionnaire, eight owners returned two questionnaires and two owners returned completed questionnaires for three and six dogs each. Not all owners answered all the questions in the questionnaire, resulting in varying response rates for different variables. Case records were retrieved from clinics in eight dogs. The final study population consisted of 52 males and 53 females. Summary statistics for the dogs included in the study are presented in Table 1.

**Table 1:** Dog characteristics, number of dogs with dilated cardiomyopathy (DCM) and pneumonia in Irish Wolfhounds born 2006–2008

Group	2006	2007	2008
Number of dogs	45	39	21
Response rate	41%	40%	30%
Alive/dead	9/36 (45)	11/28 (39)	9/12 (21)
Sex, male/female	24/21 (45)	19/20 (39)	9/12 (21)
Mean weight (kg)	68 (37)	67 (36)	68 (19)
Neutered	4 (39)	8 (36)	5 (20)
Coat color, B/RB/DB/F/W/Bl	28/1/0/3/6/7 (45)	27/2/1/4/3/2 (39)	16/0/1/1/1/2 (21)
Number with offspring	7 (45)	6 (38)	5 (20)
DCM	9 (45)	7 (39)	2 (21)
Pneumonia	14 (38)	14 (36)	6 (19)

Total number of values for each specific category is presented in parenthesis. Dogs that had been diagnosed with both DCM and pneumonia appear in both disease groups

Annual response rates; 2006: 45 answers from 109 distributed questionnaires; 2007: 39 answers from 98 distributed questionnaires; 2008: 21 answers from 69 distributed questionnaires

*B* coat color brindle, *RB* red brindle, *DB* dark brindle, *F* fawn, *W* wheaten, *Bl* black

### Causes of death

**Fig. 1:**
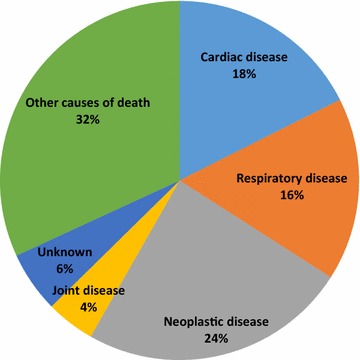
Proportional mortality for the 76 dead Irish Wolfhounds

Of the 105 dogs included in the study, 76 dogs were dead. Two of them had undergone a post mortem examination. Thirteen owners had reported multiple causes of death for their dog, which all were accounted for in the statistical analysis. The most common causes of death were neoplasia, cardiac disease and respiratory disease (Fig. 1). Neoplasia accounted for 24% of the cases of death, where osteosarcoma represented 9% and remaining 15% was neoplasia of unspecified type. Dogs having cardiac disease as cause of death included dogs classified to have DCM (17%) and unspecified cardiac disease (1%). All dogs with respiratory disease as cause of death had pneumonia. Remaining causes of death included a variety of diseases, namely gastric dilation volvulus (6.6%), megaesophagus (3.3%), paresis (3.3%), epilepsy (2.2%), pyometra (2.2%), kidney disease (2.2%), lethargy (2.2%), osteomyelitis or septic arthritis (2.2%) and 1.1% for the following causes; diabetes mellitus, traffic accident, intoxication, behavioral problems, stiffness of the neck, skeletal pain and old age.

Of the deceased dogs, all but five dogs had been euthanized. The remaining five dogs had died suddenly, which result in a sudden death rate of 7%. One of these dogs had undergone a post mortem examination, and was diagnosed with acute circulatory failure with a strong suspicion of DCM. Another dog experiencing sudden death was diagnosed with DCM prior to sudden death, and for the remaining dogs the owners suspected gastric dilation volvulus in one case, and no specific disease was suspected as cause of death in the remaining two dogs.

### Dilated cardiomyopathy

Overall the owner-reported frequency of DCM in the study was 17% (18 dogs). For a majority of these dogs (n = 14), owners had declared their dog to be diagnosed with DCM, and for two dogs the diagnosis were also confirmed by post mortem examination. The remaining dogs (n = 4) were classified as probably affected by DCM (n = 2), or with a strong suspicion of DCM (n = 2). The proportion of dogs affected by DCM among males was 21% and among females 13%, but no statistical difference between sexes was found (P = 0.31).

### Pneumonia

The proportion of dogs with owners responding that the dog had pneumonia on at least one occasion was 37% (n = 34). The response for this question included 93 answers. The proportion of male and female dogs with at least one episode of pneumonia was 37 and 36%, respectively. There was no significant difference in proportions of dogs with a history of pneumonia between sexes (P = 1.00).

Among dogs with a history of pneumonia, all 34 owners had answered the question regarding recurrence. Eighteen dogs (53%) had recurrent episodes of pneumonia and 11 of them had developed pneumonia three times or more, and two dogs were reported to have developed pneumonia 14 times during their lifetimes.

In the 34 dogs with at least one episode of pneumonia, 15% (n = 5) had also been diagnosed with DCM, and for one of these dogs, the owner had reported the dog to have developed pneumonia 14 times during its lifetime. In three of the 34 dogs, the owners had also reported megaesophagus as a concurrent disease. Two of these dogs had been diagnosed with megaesophagus by radiographic examination, but no information was provided how the diagnosis was established in the third dog. Of the 28 deceased dogs with at least one episode of pneumonia, 50% had pneumonia reported as a cause of death.

### Lifespan

**Fig. 2:**
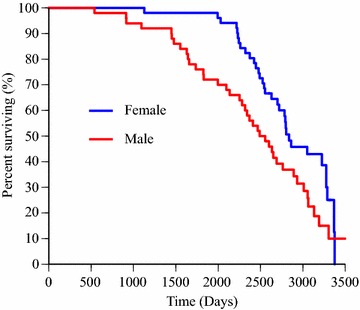
Gender-based survival in 101 Irish Wolfhounds. Kaplan–Meier curves for dogs divided into groups based on sex. Males had significantly shorter lifespan than females (P < 0.05). Right censored data was included and four dead dogs were excluded because no date of death was reported

**Fig. 3:**
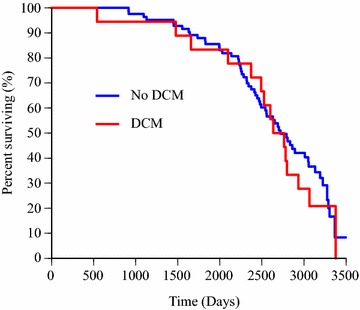
Survival in 18 Irish Wolfhounds with dilated cardiomyopathy (DCM) and in dogs without DCM (n = 83). Kaplan–Meier curves for dogs divided into subgroups with or without a diagnosis of DCM. The difference in lifespan was not significant. Right censored data was included and four dead dogs were excluded because no date of death was reported

The median lifespan in IW participating in this study is presented in Table 2. Kaplan–Meier survival curves for male and female dogs are presented in Fig. 2, showing that males had a shorter lifespan than females (P = 0.02). Kaplan–Meier survival curves for dogs with and without DCM are shown in Fig. 3, (P = 0.64). The effect of sex on survival was maintained in the multivariable Cox proportional hazard analysis (P = 0.02), which included sex and DCM diagnosis (yes/no). The latter variable remained insignificant on the outcome. Hazard ratios for the variables in the multivariable Cox proportional hazard analysis are presented in Table 3.

**Table 2:** Median lifespan and interquartile range in days for the entire study population and subgroups

Group	Median lifespan	Interquartile range	Number of responses
Deceased dogs	2477	792	73
Entire study population^a^	2720	1014	101
Males^a^	2523	1238	50
Females^a^	2836	890	51
DCM^a^	2699	694	18
No DCM^a^	2720	1023	83
Pneumonia^a^	2629	790	34
No pneumonia^a^	2804	985	56

Dead dogs, in which no date of death was reported; were excluded, as were dogs in the no pneumonia subgroup, if the owner had not provided an answer regarding occurrence of pneumonia

*DCM* dilated cardiomyopathy

^a^Right censored data included

**Table 3:** Hazard ratios for the variables sex and a diagnosis of dilated cardiomyopathy (DCM) in the multivariable analysis

Variable	Hazard ratio	95% CI	P value
Male (yes/no)	1.73	1.08–2.77	0.02
DCM (yes/no)	1.17	0.64–2.01	0.60

Result from the multivariable Cox proportional hazard analysis with the variables sex and a diagnosis of dilated cardiomyopathy (DCM), with 95% confidence intervals (95% CI) and P values

**Fig. 4:**
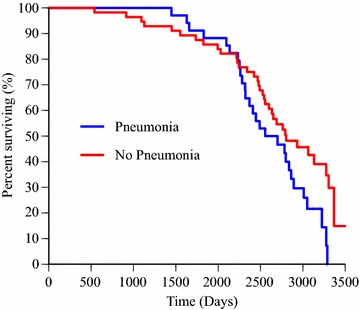
Survival in 34 Irish Wolfhounds with history of pneumonia and in dogs (n = 56) without a history of pneumonia. Kaplan–Meier curves for dogs divided into subgroups with a history of pneumonia diagnosed at least on one occasion and those without history of pneumonia. The difference in lifespan was statistically significant (P < 0.05). Right censored data was included. Four dead dogs were excluded because no date of death was reported and additional 11 dogs were excluded because the owner had not provided an answer regarding occurrence of pneumonia

The impact of a diagnosis of pneumonia on survival is shown in Fig. 4. Dogs with history of pneumonia on at least one occasion had shorter lifespan compared to those without history of pneumonia (P = 0.04), and this difference remained significant in the multivariable Cox proportional hazard analysis (P = 0.03), which included the variables sex and occurrence of pneumonia in the model. The effect of sex was maintained (P = 0.03) in this model. Hazard ratios for these variables are presented in Table 4.

**Table 4:** Hazard ratios for the variables sex and a diagnosis of pneumonia in the multivariable analysis

Variable	Hazard ratio	95% CI	P value
Male (yes/no)	1.74	1.05–2.90	0.03
Pneumonia (yes/no)	1.81	1.07–3.03	0.03

Result from the multivariable Cox proportional hazard analysis with the variables sex and a diagnosis of pneumonia on at least one occasion, with 95% confidence intervals (95% CI) and P values

## Discussion

In this study, we found a high proportion (37%) of pneumonia among the IW and a significant shorter lifespan in dogs affected by pneumonia, the latter has not previously been reported. Most common causes of death among IWs were neoplastic and cardiac diseases along with respiratory disease. The study also showed that females lived longer than males and that the proportion of DCM reported by the owners was high (17%), but the effect of DCM on lifespan was not significant.

### Cause of death

Results from this study, showing neoplastic and cardiac disease to be the most common causes of death, are supported by previous studies based on Swedish insurance data [1, 20], where the breed was found to have an increased mortality rate for cardiac- and neoplastic disease. In the present study, we found a high proportion of deaths due to pneumonia, which is in agreement with a study by Fleming et al. [3] who reported respiratory disease to be the top four most common causes of death in North American IW.

Neoplastic disease was not the main focus of the present study, but was the most common cause of death and other studies have also reported neoplastic disease to be over-represented as cause of death in the breed [2, 3]. In the present study osteosarcoma accounted for a large portion of all neoplastic diseases, and other studies have also shown bone neoplasia to have an increased incidence rate in the breed [4, 21]. Furthermore, musculoskeletal disorders have previously been reported as a common cause of death in IW [1, 3]. In the study by Egenvall et al. [1], osteosarcoma was not included in musculoskeletal diseases, but in the study by Fleming et al. [3] musculoskeletal diseases included all diseases localized to the specific organ system which allowed neoplasias, such as osteosarcoma, to be included. In the present study, joint disease only accounted for 4% of deaths, which is a low proportion compared to previous studies [3, 20]. However, some owners reported other causes of death, which cannot with certainty be distinguished from musculoskeletal or neurological disorders, such as paresis and stiffness of the neck (accounted for 3% and 1% in the present study, respectively). Previous studies have shown diseases in the gastrointestinal organ system to be a common cause of death [3], and in our study, gastric dilation volvulus accounted for 7% of causes of death.

### Dilated cardiomyopathy

The owner-reported occurrence of DCM in the studied population was 17%. Among Swedish owners of IW, DCM has gained attention because it is a serious and fatal disease, and because insurance data has identified it as a major cause of death in the breed [1, 12]. This may result in an interest to participate in this study; particularly owners or breeders of dogs having died from DCM, or those highly committed to improve the general health situation of the breed, are likely to respond to surveys like the present. However, a retrospective study like this will only identify dogs diagnosed with DCM, but not dogs with preclinical DCM, unless they had undergone an echocardiographic examination. Brownlie and Cobb [11] reported that 12% of IW without clinical sign of cardiac disease had atrial fibrillation, which they suggested to be a possible indication of preclinical DCM. Therefore, the occurrence of DCM among the IW may be higher than reported in the present study (17%). There is, consequently, a major difference between the present and other studies reporting a higher occurrence of DCM (24–43%), namely that these studies included results from breed screening using echocardiography, and therefore included more cases with preclinical disease [5, 6, 13]. However, four of the dogs classified to have DCM in this study, were classified as probably affected or with suspicion of DCM by the authors based on information from the questionnaire such as presence of atrial fibrillation and/or development of CHF. Dogs with atrial fibrillation were included in the DCM group since reports suggest that atrial fibrillation appears to be associated to DCM in IW [5, 11, 22]. Nevertheless, a few individuals may have been misdiagnosed with DCM.

When comparing the occurrence of DCM by year of birth, the disease seems to be more common among dogs born 2006 and 2007 compared to 2008. This is most probably an effect of a lower response rate for dogs born in 2008, and therefore smaller sample size. Another possibility is that dogs born 2008 were younger (7 years old to current date) and had not yet developed DCM. However, studies have reported the mean age of diagnosis with DCM to be 4–5 years [5, 6, 14]. Most dogs born 2008 would therefore already have developed the disease if they would have had the predisposition. A third possibility is that the disease could be decreasing in incidence in the breed.

In the present study, there was no significant difference in occurrence of DCM between sexes, although the occurrence was 21% among males and 13% among females. A sex predisposition has been shown in a previous study where DCM in IW was more common among males than females [6]. The lack of a significant difference in the present study may be due to the small sample size, with only 18 dogs classified as having DCM.

### Pneumonia

Our study showed that 37% of the study population had a history of pneumonia and that these dogs had a significantly shorter lifespan than those without history of pneumonia. The occurrence of pneumonia was nearly equally distributed among males and females. Few studies have been published regarding pneumonia in IW. These studies also report that the incidence in the breed is high and respiratory disease is frequently reported as a cause of death [3, 17]. In our study, 53% of the dogs with history of pneumonia had at least one recurrent episode. The comparably high number of dogs where the owner had reported recurrent episodes of pneumonia as well as pneumonia as cause of death, in combination with the documented reduced lifespan and increased hazard ratio, suggest that the pneumonias are severe and that respiratory disease is a serious problem in the breed. Greenwell and Brain [17] found a predisposing cause for aspiration pneumonia in 44% (n = 4) of the IW in their study. In our study, three of the dogs had megaesophagus (two diagnosed by radiology, in the third dog no information was provided on how the diagnosis was established), which may suggest this abnormality as cause for pneumonia in these dogs. Only 15% of the dogs (n = 5) with history of pneumonia had also been diagnosed with DCM. Nevertheless, a possible association between pneumonia and DCM would be of interest to explore further in the future.

### Lifespan

Median lifespan in IW participating in this study were 2720 days (7.5 years), which is longer than previous results in studies from Norway and Great Britain [2, 4]. Dogs with history of pneumonia lived shorter than those without such a history. Our result regarding median lifespan for IW with cardiac disease (7.4 years), is similar to previous studies of Swedish insurance data (7.5 years) [12]. A study concerning age of onset of CHF due to DCM in IW have shown a mean age of 6.4–7.2 years [11], and survival after development of CHF in dogs of different breeds with DCM was only 18% after 1 year [8]. These results are in agreement with ours regarding lifespan in IW with DCM (7.4 years), even though the study by Tidholm et al. [8] included data from dogs of different breeds, and prognostic differences between breeds exist [9, 11]. Moreover, in the present study, data is based on dogs, which likely have been treated with pimobendan, a cardiac drug, which has been shown to significantly increase the lifespan in dogs with DCM [23, 24]. A recent study did show pimobendan to significantly prolong time to CHF or sudden death in IW with preclinical DCM or atrial fibrillation compared to other cardiac drugs [25]. This means that results from the present study, regarding survival among dogs affected by DCM, cannot completely be compared with previous older studies, where dogs were not treated with pimobendan. Moreover, effect of CHF therapy, and course of treatment of pneumonias, have not been evaluated in the present study. They are likely to have affected survival.

### Study limitations

The survey had a response rate of 38% corresponding to 105 dogs, which is a rather small sample size and results in limitations regarding detection of clinically important differences. Furthermore, the small sample size impacts the number of variables that can be included in the statistical models. The response rate in this study is comparable to other similar studies, with small differences in study design, with reported response rates of 21–51% [4, 26–28], and suggests the response rate in this study to be reasonable.

The study population included a cohort of IW born during 2006–2008. During this period 460 dogs were born and registered in the SKK, but for 180 dogs no contact details were available, and these dogs were excluded. Hence, only a minority of the dogs born during these years were included in the study population, and the results may not entirely be representative of the general Swedish IW population. There are, however, no indications that included dogs were biased in either direction with regards to presence and severity of disease. The years were specifically chosen to allow studies of morbidity, mortality and lifespan, because the dogs had reached an age (7–9 years) where many events may have occurred, but not too far back in time for the owner to forget details of their dog’s health situation. The latter represents one important limitation with retrospective survey studies, because important details about clinical signs, cause of death and date of death might have been forgotten or mixed up with other dogs in the household. When a retrospective survey, like the present, is performed, information concerning clinical signs and diagnoses are based on the owners’ perceptions and knowledge about type of examinations that were performed. Naturally, this is very dependent on how good the communication between owner and veterinarian has been. However, an advantage with a survey compared to clinical case series including dogs visiting veterinary hospitals, is that the results will not be skewed by selection bias. Dogs attending veterinary clinics are more likely to be sick, which would lead to higher prevalence of disease.

Regarding dogs with pneumonia, insufficient information was available to determine how the dogs had been diagnosed and how they had been treated. The results were only based on the information provided by the owner, which, in turn, is dependent on how attentively and vigorously owners monitored health status of their dogs. Questions were asked about course of treatment, time of recurrence and if full recovery was made, but these questions were often answered inadequately, and did not allow any further analysis. The consequence of owner reported diagnoses in this study might have resulted in slightly overrepresented occurrence of pneumonias, depending on the owners’ perceptions and interpretation of respiratory clinical signs. However, the presence of DCM was probably more reliable because most of these dogs had undergone diagnostic imaging tests of the heart and thorax.

Regarding cause of death, only two of the deceased dogs had undergone a post mortem examination, which means that causes of death were determined with different degrees of certainty for the dogs.

## Conclusions

The most common causes of death among the IW participating in this study were neoplastic, cardiac and respiratory diseases. The owner-reported occurrence of DCM and pneumonia in the studied population was high. A high proportion of dogs with history of pneumonia had recurrent episodes. Median lifespan was significantly shorter for males than females and for dogs with a history of pneumonia compared to those without history of pneumonia.

Pneumonia, DCM and osteosarcoma are major health problems in IW dogs. The impact of these diseases on the general health in the breed should raise concerns amongst breeders and veterinarians. The results might also provide a basis for clinical decision making and preventive measures aimed at reducing the occurrence of these diseases.

## Additional file

**Additional file 1: Questionnaire.** A translated English version of the questionnaire distributed to owners of purebred Irish Wolfhounds born during 2006–2008.

## Abbreviations

CHF: congestive heart failure
DCM: dilated cardiomyopathy
IW: Irish Wolfhound
SKK: Swedish Kennel Club
SLU: Swedish University of Agricultural Sciences
