# Supplementary material for: Occurrence of cardiorespiratory diseases and impact on lifespan in Swedish Irish Wolfhounds: a retrospective questionnaire-based study
Source: Acta Vet Scand. 2017 Aug 2;59:53. doi: 10.1186/s13028-017-0320-1 (PMC5540459; doi:10.1186/s13028-017-0320-1)
Supplement: Supplementary file 1 — Additional file 1: Questionnaire. A translated English version of the questionnaire distributed to owners of purebred Irish Wolfhounds born during 2006–2008. [file 13028_2017_320_MOESM1_ESM.pdf]

Survey

- a health evaluation of Irish wolfhounds

<u>All information about You and Your dog will be treated confidentially</u>

*Mandatory questions are marked with **

Part 1

Information about the dog

**Reg. no: ***	**Breed:**<br>**Irish wolfhound**	
**Registered name: ***	**ID: chip and/or tattoo:**	
**The dog's name:**	**Date of birth: ***	**Coat color:**
**Sex ***<br><br>□ Male<br>□ Female	**Neutered or spayed ***<br><br>□ Yes Date:______________<br>□ No	
Weight _____ kg<br>□ Normal weight □ Under normal weight □ Overweight		
**Has the dog been used for breeding?**<br>□ Yes □ No<br>If Yes, how many offsprings does it have? ___________________________<br><br>**Have relatives to the dog been diagnosed with dilated cardiomyopathy?**<br>*(Parents, siblings and offspring are defined as relatives)*<br>□ Yes □ No<br>If Yes, which? ____		

Owner

**First name: ***	**Surname: ***	**Country: ***
**Street: ***	**Postal code: ***	**City: ***
**e-mail:**	**Telephone:**<br>Home:<br>Cell phone:<br>Office:	

Continue to part 2 if the dog is DEAD or to part 3 if the dog is ALIVE

PART 2: The dogs is DEAD

How did the dog die *

Euthanasia ……………………………………… □
Spontaneously ……………..……………… □
Other………………………………………… □
If Other, specify: ____

Date of death/euthanasia: * ____________________

Cause of death *

Dilated cardiomyopathy (congestive heart failure) …....... □
Other heart disease ………………………………………………. □
Bone cancer (osteosarcoma)……………..……………… □
Other tumor ………………………………………… □
Pneumonia ………………………………………… □
Joint disease ……………………………………... □
Unknown cause ……………………................................... □
Other ………………………………………………………………. □
If Other, specify: ____

The diagnosis was made by *

Echocardiography ………............…………………… □
Radiography ………………………................................. □
Necropsy ………………………...................................... □
Unknown ………………………....................................... □
Other ………………………………………………………………. □
If Other, specify: ____

Was the dog diagnosed with dilated cardiomyopathy prior to death? *

□ Yes □ No
If Yes, approximate date: ____

If Yes, did the dog receive any medical treatment due to heart disease?

□ Yes □ No

Specify which drugs: ____

If your dog was diagnosed with dilated cardiomyopathy, did the veterinarian diagnose congestive heart failure (fluid in the lungs and/or abdomen)?

□ Yes □ No □ Don't know

Did the dog have clinical signs prior to death? *

□ Yes □ No
If Yes, specify by checking below.

Clinical signs

Heart murmur ……………………………........................... □
Irregular heart rhythm/arrhythmia ……………..…….……. □
If arrhythmia, specify which type: ______________________________
Fatigue ………….......................………….......................... □
Exercise intolerance ………………………………….…… □
Laboured breathing………….........……………………… □
If laboured breathing, describe breathing pattern: ____________________

Cough ………..………. □
Abdominal distention ……..........……………………………… □
Poor appetite …………...……….… □
Weight loss …………...................................………………..□
Other …………...……… □
If Other, specify: ____

Was necropsy performed? * □ Yes □ No

Diagnosis from necropsy
Dilated cardiomyopathy ……………………………………..…..….□
Tumor ………….. □
Pneumonia ……….……..……………………………..…… □
Joint disease ……..□
Trauma ………..………...…... □
Unknown …………………………..................................….... □
Other ………….. □
If Other, specify: ____

Has the dog ever been diagnosed with pneumonia? *

□ Yes □ No
If Yes, approximate date: ______________________________________

How long was the dog treated for pneumonia?____________________________
Did the dog recover? □ Yes □ No

Did the dog have pneumonia on more than one occasion?
□ Yes □ No
If Yes, specify how many occasions and with approximate dates: ________________________

Have relatives to the dog been affected by pneumonia? *

(Parents, siblings and offspring are defined as relatives)
□ Yes □ No If Yes, which? ____________________________

Information about the veterinarian responsible for treatment

Name of veterinarian: ____

Name of veterinary clinic: ____

Address: ____

Postal code, city and country: ____

Telephone: _________________________________ e-mail: ________________

Owner gives us (J. Häggström, I. Ljungvall, K. Höglund eller L. Orleifson) permission to access the dog's case records*:*

□ Yes □ No

Thank you for your participation!

PART 3: The dog is ALIVE

Owner's description of the dog *

Healthy………………………………………………………………………………………… □
Dilated cardiomyopathy, untreated, no clinical signs from the heart .... □
Dilated cardiomyopathy, receiving medical treatment but have never had clinical signs from the heart……………………………………………………………………………………… □
Dilated cardiomyopathy, receiving medical treatment and have had clinical signs from the heart .... ………………………………………………………. □
Other ……………………………………………………………………………………………… □
If Other, specify: ____

Clinical signs

Heart murmur …………………………………………………… □
Irregular heart rhythm/arrhythmia ……………………………… □
If arrhythmia, specify which type: ________________________________
Fatigue ……………………………………………………………… □
Exercise intolerance ………………………………………………… □
Laboured breathing ………………………………………………… □
If laboured breathing, describe breathing pattern: ____________________
Cough ……………………………………………………………… □
Abdominal distention ………………………………………………… □
Poor appetite ……………………………………………………… □
Weight loss ………………………………………………………… □
Other ……………………………………………………………… □
If Other, specify: ____

Veterinary examination has been performed *

□ Yes □ No

Date: ______________________

Information about the veterinarian responsible for treatment

Name of veterinarian: ____

Name of veterinary clinic: _____________________________________

Address: ____

Postal code, city and country: __________________________________

Telephone: ___________________________ e-mail: ______________

Owner gives us (J. Häggström, I. Ljungvall, K. Höglund eller L. Orleifson) permission to access the dog's case records:

□ Yes □ No

Information from the veterinary examination

Echocardiography has been performed *
□ Yes □ No

Diagnosis from the echocardiography examination
Dilated cardiomyopathy ....□
Congenital heart defect ....□
Other heart disease ……………..□
Other...□
If Other heart disease or Other, specify: ________________________________

Thoracic radiographs have been taken *
□ Yes □ No

Diagnosis from the radiography examination
Pulmonary edema/fluid in the lungs................ □
Heart enlargement……………………………..... □
Pneumonia ……………………….…..….…..….□
Other………………………………………....…..… □
If Other, specify: ____

If your dog has been diagnosed with dilated cardiomyopathy, did the veterinarian diagnose congestive heart failure (fluid in the lungs and/or abdomen)?
□ Yes □ No □ Don't know

Does the dog receive any medical treatment due to heart disease? *
□ Yes □ No

Specify which drugs: ____

When did the medical treatment start; date: ______________________________

Outcome of treatment
No response ……………□
Improved ……………….□
Good response ……..….□
Other ……………………□
If Other, specify: ____

Has the dog ever been diagnosed with pneumonia? *
□ Yes □ No
If Yes, approximate date: ____

How long was the dog treated for pneumonia?__________________________
Did the dog recover? □ Yes □ No

Did the dog have pneumonia on more than one occasion?

□ Yes □ No

If Yes, specify how many occasions and with approximate dates: ________________________

Have relatives to the dog been affected by pneumonia? *

(Parents, siblings and offspring are defined as relatives)

□ Yes □ No If Yes, which? ____________________________

Thank you for your participation!
